# Supplementary material for: Hybrid Models and Biological Model Reduction with PyDSTool
Source: PLoS Comput Biol. 2012 Aug 9;8(8):e1002628. doi: 10.1371/journal.pcbi.1002628 (PMC3415397; doi:10.1371/journal.pcbi.1002628)
Supplement: Text S4 — Complete source code for the PyDSTool package (version 0.88.120504). Includes API documentation and help files linking to web pages. This file is identical to the current public release on Sourceforge.net. (ZIP) [file pcbi.1002628.s004.zip › PyDSTool/html/toc-PyDSTool.ModelSpec'-module.html]

xml version="1.0" encoding="ascii"?


ModelSpec'


# Module ModelSpec'

---

## Classes

Component  
 LeafComponent  
 ModelSpec  
 nameResolverClass  

regObject

typeCounter

## Functions

accrueCompTypes

allCompTypes

matchSubName  
 processMacro  
 resolveMacroTargets  
 searchModelSpec  

## Variables

API

Abs

Acos

Asin

Atan

Atan2

Betavariate

Ceil

Choice

Continuous

Cos

Cosh

Degrees

Discrete

E

Exp

Expovariate

Fabs

Floor

Fmod

Frexp

Gammavariate

Gauss

Getrandbits

Getstate

Hypot

Inf

Jumpahead

LargestInt32

Ldexp

Log

Log10

Lognormvariate

Max

Min

Modf

NAMESEP

NaN

Normalvariate

ONES

Paretovariate

Pi

Pow

Radians

Randint

Random

Randrange

Sample

Seed

Setstate

Shuffle

Sin

Sinh

Sqrt

Sum

Systemrandom

Tan

Tanh

Uniform

Vonmisesvariate

Weibullvariate

Wichmannhill

ZEROS

\_1DimplicitSolveMethods

\_all\_complex

\_all\_float

\_all\_int

\_all\_numpy\_complex

\_all\_numpy\_float

\_all\_numpy\_int

\_classes

\_complex\_types

\_float\_types

\_functions

\_implicitSolveMethods

\_indentstr

\_int\_types

\_num\_equivtype

\_num\_maxmin

\_num\_name2equivtypes

\_num\_name2type

\_num\_type2name

\_num\_types

\_objects

\_pytypefromtype

\_real\_types

\_seq\_types

allmathnames

allmathnames\_symbolic

builtinFnSigInfo

builtin\_auxnames

e

inverseMathNameMap

isfinite

mathNameMap

mod

nameResolver  

name\_chars\_RE

null\_predicate

num\_chars

pi

protected\_allnames

protected\_auxnamesDB

protected\_macronames

protected\_mathnames

protected\_randomnames

protected\_scipynames

protected\_specialfns

specTypes

syms

targetLangs

---

[hide private]
